# Supplementary material for: Pharmacokinetic Outcomes of the Interactions of Antiretroviral Agents with Food and Supplements: A Systematic Review and Meta-Analysis
Source: Nutrients. 2022 Jan 25;14(3):520. doi: 10.3390/nu14030520 (PMC8840371; doi:10.3390/nu14030520)
Supplement: Supplementary file 1 [file nutrients-14-00520-s001.zip › supplementary material S1_search terms.pdf]

Supplementary material S1

Table S1. Search terms for study screening.

**Databases**	**Search terms**
**Cochrane**	("dietary supplement" OR "food supplement" OR "nutrient supplement" OR "nutritional supplement" OR vitamin OR mineral OR calcium OR protein OR zinc OR magnesium) AND ("Antiretroviral Therapy, Highly Active" OR HAART OR "antiretroviral therapy" OR "antiretroviral drug" OR "non-nucleoside reverse transcriptase inhibitor" OR "protease inhibitor" OR "integrase inhibitor" OR bictegravir OR tenofovir OR emtricitabine OR dolutegravir OR lamivudine) AND ("area under curve" OR "area under curve" OR AUC OR "plasma concentration" OR Cmax OR Cmin OR (("Cytochrome P-450 Enzyme System" OR "cytochrome P450" OR "cytochrome P 450" OR "cytochrome P-450" OR CYP450 OR CYP OR "ATP Binding Cassette Transporter, Subfamily B" OR "p-glycoprotein" OR "p glycoprotein" OR PGY1 OR PGY-1) AND (inducer OR induction OR inhibitor OR inhibition)))
**ScienceDirect**	((("dietary supplement" OR "food supplement" OR nutrient) AND ("antiretroviral therapy" OR "antiretroviral drug") AND ("area under curve" OR "plasma concentration" OR CYP)))
**Scopus**	"(TITLE-ABS-KEY("dietary supplement" OR "food supplement" OR "nutrient supplement" OR "nutritional supplement" OR vitamin OR mineral OR calcium OR protein OR zinc OR magnesium)) AND (TITLE-ABS-KEY ("antiretroviral therapy, highly active" OR HAART OR "antiretroviral therapy" OR "antiretroviral drug" OR "triple therapy" OR "non-nucleoside reverse transcriptase inhibitor" OR "protease inhibitor"

	OR "integrase inhibitor" OR bictegravir OR tenofovir OR emtricitabine OR dolutegravir OR lamivudine)) AND (TITLE-ABS-KEY ("area under curve" OR AUC OR "plasma concentration" OR Cmax OR Cmin OR "cytochrome P-450 enzyme system" OR "cytochrome P450" OR "cytochrome P 450" OR CYP450 OR CYP OR "ATP Binding Cassette Transporter, Subfamily B, Member 1" OR "p-glycoprotein" OR "p glycoprotein" OR PGY1)) AND (TITLE-ABS-KEY (inducer OR induction OR inhibitor OR inhibition))"
**PubMed**	("dietary supplements"[Mesh] OR "dietary supplement"[tw] OR "food supplement"[tw] OR "nutrient supplement"[tw] OR "nutritional supplement"[tw] OR vitamin[tw] OR mineral[tw] OR calcium[tw] OR protein[tw] OR zinc[tw] OR magnesium[tw]) AND ("Antiretroviral Therapy, Highly Active"[Mesh] OR HAART[tw] OR "antiretroviral therapy"[tw] OR "antiretroviral drug"[tw] OR "non-nucleoside reverse transcriptase inhibitor"[tw] OR "protease inhibitor"[tw] OR "integrase inhibitor"[tw] OR bictegravir[tw] OR tenofovir[tw] OR emtricitabine[tw] OR dolutegravir[tw] OR lamivudine[tw]) AND ("area under curve"[Mesh] OR "area under curve"[tw] OR AUC OR "plasma concentration"[tw] OR Cmax[tw] OR Cmin[tw] OR (("Cytochrome P-450 Enzyme System"[Mesh] OR "cytochrome P450" [tw] OR "cytochrome P 450"[tw] OR "cytochrome P-450"[tw] OR CYP450[tw] OR CYP[tw] OR "ATP Binding Cassette Transporter, Subfamily B, Member 1"[Mesh] OR "p-glycoprotein"[tw] OR "p glycoprotein"[tw] OR PGY1[tw] OR PGY-1[tw]) AND (inducer[tw] OR induction[tw] OR inhibitor[tw] OR inhibition[tw])))
