# Supplementary material for: Pharmacokinetic Outcomes of the Interactions of Antiretroviral Agents with Food and Supplements: A Systematic Review and Meta-Analysis
Source: Nutrients. 2022 Jan 25;14(3):520. doi: 10.3390/nu14030520 (PMC8840371; doi:10.3390/nu14030520)
Supplement: Supplementary file 1 [file nutrients-14-00520-s001.zip › supplementary material S2_quality assessment.pdf]

**Supplementary material S2**

**Table S2.** The quality assessment of included studies.

Case report

| Author, year | Selection | Ascertainment of exposure | Ascertainment of outcome | Alternative causes | Rechallenge | Dose response | Follow-up duration | Sufficient reporting | Quality of study |
|---|---|---|---|---|---|---|---|---|---|
| Roberts 2011 | Yes | Yes | Yes | No | Yes | Yes | Yes | Yes | Good |

Cross-sectional study

| Author, year | Representativeness of the sample | Sample size | Non-respondents | Ascertainment of the exposure | Confounders are controlled | Assessment of the outcome | Statistical test | Quality of study |
|---|---|---|---|---|---|---|---|---|
| Daskapan 2017 | * | * | * | ** | - | * | * | Fair |

Poor (1-3)*
Fair (4-6)*
Good (7-10)*

Randomized controlled trial

| Author, year | Random sequence generation | Allocation concealment | Blinding of participants and personnel | Blinding of outcome assessment | Incomplete outcome data addressed | Selective reporting | Other bias | Quality of study |
|---|---|---|---|---|---|---|---|---|
| Jensen-Fangel 2003 | + | + | - | - | ? | + | ? | Fair |
| Abdissa 2015 | + | + | + | ? | + | - | - | Fair |

| | | | | | | | | |
|---|---|---|---|---|---|---|---|---|
| Munkombwe 2016 | + | + | - | - | ? | ? | ? | Fair |

+ low risk
- high risk
? unclear risk

Cross-over study

| Author, year | Appropriate cross-over design | Randomized treatment order | Carry-over effect | Unbiased data | Allocation concealment | Blinding | Incomplete outcome data | Selective outcome reporting | Other bias | Quality of study |
|---|---|---|---|---|---|---|---|---|---|---|
| Kupferschmidt 1998 | + | - | ? | ? | ? | - | + | + | ? | Fair |
| Carver 1999 | + | - | ? | ? | ? | - | + | + | ? | Fair |
| Moore 1999 | + | + | + | ? | ? | - | + | + | ? | Good |
| Yuen 2001 | ? | + | ? | ? | ? | - | + | + | ? | Good |
| Penzak 2002 | + | + | ? | ? | ? | - | ? | ? | ? | Fair |
| Falcoz 2002 | + | + | ? | ? | ? | - | ? | + | ? | Fair |
| Mouly 2005 | + | + | ? | ? | ? | ? | + | ? | ? | Fair |
| Sekar 2007 | + | + | ? | ? | ? | - | + | + | ? | Good |
| Patel 2011 | + | + | ? | ? | ? | - | + | + | ? | Good |
| Song 2015 | + | + | ? | ? | ? | - | + | + | ? | Good |
| Buchanan 2017 | + | + | ? | ? | ? | - | + | + | ? | Good |

| | | | | | | | | | | |
|---|---|---|---|---|---|---|---|---|---|---|
| Yamada 2018 | + | + | ? | ? | ? | - | + | + | ? | Good |
| Yonemura 2018 | + | + | ? | ? | ? | - | + | + | ? | Good |

+ low risk
- high risk
? unclear risk

Longitudinal study

| Author, year | Selection of participants | Classification of intervention | Deviation from intended intervention | Missing data | Measurement of outcome | Selection of reported result | Confounding factors addressed | Quality of study |
|---|---|---|---|---|---|---|---|---|
| Piscitelli 2002 | + | + | ? | - | + | + | ? | Good |
| Slain 2005 | + | + | ? | + | + | + | ? | Good |

+ low risk
- high risk
? unclear risk

Pharmacokinetic study

| Author, year | Were the criteria for inclusion in the sample clearly defined? | Were the study subjects and the setting described in detail? | Was the exposure measured in a valid and reliable way? | Were objective, standard criteria used for measurement of the condition? | Were confounding factors identified? | Were strategies to address confounding factors stated? | Were outcomes measured in a valid and reliable way? | Was appropriate statistical analysis used? | Quality of study |
|---|---|---|---|---|---|---|---|---|---|
| DiCenzo 2006 | + | + | + | + | ? | ? | + | + | Good |

| | | | | | | | | | |
|---|---|---|---|---|---|---|---|---|---|
| Robertson 2008 | + | + | + | ? | ? | ? | ? | ? | Fair |
| Sheehan 2012 | + | + | + | ? | ? | ? | ? | ? | Fair |
| Calderõn 2014 | + | + | + | + | ? | ? | + | + | Good |

+ low risk
- high risk
? unclear risk
